# Rapidly assessing the risks of infectious diseases to wildlife species

**DOI:** 10.1098/rsos.181043

**Published:** 2019-01-16

**Authors:** Wendy Beauvais, Steffen Zuther, Chantal Villeneuve, Richard Kock, Javier Guitian

**Affiliations:** 1 Royal Veterinary College, Hatfield, UK; 2 Cornell University College of Veterinary Medicine, Ithaca, NY, USA; 3 Association for the Conservation of Biodiversity of Kazakhstan, Astana, Kazakhstan; 4 Frankfurt Zoological Society, Frankfurt am Main, Germany

**Keywords:** risk assessment, risk map, livestock, wildlife, disease transmission

## Abstract

Predicting the likelihood of rare events is increasingly demanded by risk managers. A key challenge is dealing with different types of uncertainty, including epistemic uncertainties (lack of knowledge), stochasticity (inherent randomness) and natural variation. One potentially catastrophic event which is impacted by high levels of all three of these uncertainty types is the transmission of livestock pathogens to wildlife, particularly for endangered species. There is often a lack of basic information, e.g. about a given pathogen's presence in local livestock populations or the susceptibility of a given wildlife species to infection by the pathogen. We adapted the OIE (World Organisation for Animal Health) risk assessment framework to rapidly assess and prioritize the risks of livestock pathogens for wildlife, taking account of epistemic uncertainties, stochasticity, seasonal movement of animals and interaction between different species at different spatial and temporal scales. We demonstrate the approach using the endangered saiga antelope (*Saiga tatarica tatarica*) as a case study. We conclude that, in general, transmission events are likely to be rare and limited to small geographical areas; however, their impact could be high. *Brucella* spp. and foot-and-mouth disease virus are among those most likely to be transmitted from livestock to the Betpak-Dala saiga population.

## 1. Introduction

Predicting the likelihood of catastrophic or rare events is increasingly in demand by risk managers. For example, large amounts of funding have been allocated to predicting the risks of emergence of zoonotic diseases with epidemic potential [1]. A key challenge in this process is dealing with different types of uncertainty, for example epistemic uncertainties (lack of knowledge), stochasticity (inherent randomness) and natural variation, often driven by spatial and temporal determinants [2]. Natural variation is not always classified as a type of uncertainty, but it produces uncertainties because it can lead to difficulties in synthesizing information from disparate sources to produce guidelines that are comprehensible and useful for risk managers. There is a demand for systematic assessments of the likelihood of events that limit bias and make epistemic uncertainties, stochasticity and natural variation explicit [2].

One example of a potentially catastrophic event which is impacted by high levels of epistemic uncertainty, stochasticity and natural variation is the transmission of livestock pathogens to wildlife, particularly for endangered species. Infectious diseases can potentially contribute to the extinction and endangerment of wildlife species, in combination with other factors [3]. There are also examples of domestic livestock pathogens causing mortality in wildlife. For example, deaths in ibex (*Capra sibirica*) were reportedly caused by transmission of peste des petits ruminant virus from goats in Pakistan [4] and saiga, ibex, bharal (*Pseudois nayaur*) and goitered gazelle (*Gazella subgutturosa*) from goats or sheep in Mongolia [5]. Transmission from wildlife to livestock can also be a problem. For example, in Yellowstone National Park brucellosis, a bacterial disease that causes abortion but can be controlled in livestock through vaccination or test-and-slaughter, periodically spreads from elk (*Cervus elaphus*) to cattle [6]. These types of events can negatively impact wildlife conservation efforts.

A pathogen that causes severe disease in domestic livestock may cause very few symptoms in wildlife species. However, sub-clinical infection (infection without obvious symptoms) could still be a problem, for example sub-clinical infections could lower fertility rates. Wildlife may also spread the infection further, which is a problem for both wildlife conservationists *and* farmers, because it affects public attitudes towards wildlife management (e.g. poaching) and financial investment in conservation. For example, badgers (*Meles meles*) have been controversially blamed for the failure of the bovine tuberculosis control programme in the UK [7].

In the face of a disease outbreak, there is often a fundamental lack of information about rare species or remote ecological environments, and there may be no published data whatsoever (or the available data may be vulnerable to publication bias), e.g. about a given pathogen's presence in local livestock populations or the susceptibility of a given wildlife species to infection by the pathogen. There are rarely sufficient data to quantitatively model the risks of pathogen transmission and subsequent disease events. Often, experts in specific pathogens are not experts in endangered species or their habitat, and vice versa. Stochasticity is an important feature because the occurrence of the necessary degree of contact between livestock and wildlife for pathogen transmission may be rare. The likelihood of pathogen transmission is also subject to variation over time and space, e.g. seasonal migration may affect contact between species; seasonal weather may affect the survival of pathogens in the environment, etc. For pathogens with complex life cycles, such as parasites relying on intermediate hosts, the environmental requirements may be highly specific.

Despite epistemic uncertainties, stochasticity and natural variation, decisions about managing the risks of infectious diseases often must be made rapidly. There is a demand for systematic knowledge synthesis, which limits bias and makes uncertainties and natural variation explicit. Smith *et al*. [8] highlighted the importance of ‘combining data with theory to discern the circumstances under which infectious disease is most likely to serve as an agent of extinction’. Other outcomes may also be important to consider, e.g. zoonotic diseases that may spread to wildlife researchers and diseases that may be spread to livestock via wildlife.

There are a range of measures that can be taken to mitigate risks, including: providing incentives to farmers for reporting disease; developing systems for alerting wildlife authorities about livestock disease outbreaks; developing and producing vaccines and diagnostic tests; disinfection and quarantine of livestock; management of seasonal pastures for livestock and designation of livestock exclusion zones. Understanding the risks associated with different diseases and situations helps to prioritize which pathogens are a concern, which mitigation measures are appropriate, and how to allocate resources accordingly.

Risk has been described as having two components: (i) the likelihood that a given hazard will occur, and (ii) the severity of the consequences if the hazard does occur [9]. A risk assessment aims to identify and characterize risks [10]. Risk management is a structured approach to providing the best possible judgements on risks by making use of all available information while simultaneously making uncertainties explicit [10]. More specifically, risk management has been defined as the process of identifying, characterizing, communicating and deciding what to do about a given risk.

In veterinary medicine, risk management has been well developed as a tool for minimizing the risk of exotic diseases being introduced via trade of livestock or livestock products. The World Organisation for Animal Health (OIE) framework for risk assessment is accepted as a valid scientific method to be used for trade negotiations. The framework includes defining risk pathways composed of risk steps which are assigned conditional probabilities that may be quantitative or qualitative. This approach has been modified for use in wildlife in a range of different ways [11].

Our approach is to adapt and apply the OIE framework [12] to the likelihood of pathogen transmission between livestock and wildlife. We classify the likelihood of these events occurring using all available data and expert opinion, implicitly considering stochastic effects. We make epistemic uncertainties in the likelihoods explicit by classifying the level of uncertainty using a semi-quantitative scale. We also investigate the disagreement between experts and the sensitivity of our model to expert opinion. We explicitly account for natural variation at different spatial and temporal scales, incorporating mapping and stratifying likelihoods and uncertainties by season. We account for seasonal movements of animals, physical interactions between different species at different spatial and temporal scales, mechanisms of transmission of different pathogens and seasonal effects on transmissibility. We demonstrate an application of the framework using the case of the critically endangered saiga antelope in Kazakhstan.

We used a semi-quantitative (as opposed to purely qualitative or quantitative) approach, meaning that we categorized likelihoods using semi-quantitative scales. However, this approach could be adapted to produce a quantitative probability of pathogen transmission and subsequent disease events if further information was available.

The saiga antelope is a critically endangered, nomadic, herding antelope found only in the steppes and deserts of Central Asia. Approximately 85% of saiga are found in Kazakhstan, and until 2015, the Betpak-Dala population was the largest of the three Kazakhstan populations with an estimate of approximately 51 700 adults in April 2017 [13]. Mass mortality events have occurred sporadically in the twentieth and twenty-first centuries [14]. In the spring of 2015, over 200 000 adult saiga in the Betpak-Dala population were officially confirmed dead [15]. Definitive evidence of the mechanism of these events remains elusive, but the cause of death was identified as haemorrhagic septicaemia caused by the bacterium *Pasteurella multocida* type B [15].

The Betpak-Dala population of saiga occupies a vast rangeland which is, in some places, shared with extensively kept horses, camels, cattle, sheep and goats. The saiga continue to migrate large distances seasonally, while domestic livestock are mainly kept in sedentary systems, although seasonal movement of livestock was common historically and still occurs in some cases. There is little published information on the risk posed to saiga from livestock pathogens [16–18]. Reports of foot-and-mouth disease affecting saiga populations during the Soviet era have been published in the Russian literature, and helminths were also reported [14]. However, there have been major changes in livestock numbers, distributions and husbandry practices since then that may affect the distribution and prevalence of disease [19].

## 2. Material and methods

We constructed a model framework based on the OIE risk assessment approach, as follows.

### 2.1. Model framework

#### 2.1.1. Defining the problem

First, the wildlife population, livestock population and pathogen(s) of interest were defined and species locations were identified. We selected the Betpak-Dala population, the historically largest population of saiga, as the focus. The specific study area was defined as the rangelands of the Betpak-Dala saiga population, derived from satellite collar data. Sheep, goats, cows, horses and camels are the most common livestock species in the area likely to share habitat with saiga, and so these were selected as the domestic species of interest.

A list of specific pathogens was compiled from (i) the OIE list of wildlife diseases published online [20] (these pathogens were selected by a panel of experts, ‘to be monitored both because of their importance for wild animals and for early warning purposes, in order to protect human and livestock health’); and (ii) the OIE-listed livestock diseases [21] (considered important for trade). From these two lists, pathogens were selected if they were considered to have a non-negligible likelihood of: (i) being present in cattle, sheep, goats, horses or camels in the rangelands of the Betpak-Dala saiga; and (ii) saiga and at least one domestic livestock species (cattle, sheep, goats, horses or camels) being susceptible based on literature review and existing knowledge about the biology of both the pathogen and livestock/wildlife species.

#### 2.1.2. Defining the likelihood question

We defined the likelihood question as follows:

What is the likelihood that each pathogen will be transmitted from domestic livestock to the Betpak-Dala saiga and that saiga are susceptible to infection (under current conditions)?

Here, the outcome of interest (i.e. exposure of saiga to the pathogen and susceptibility to the pathogen) does not necessarily mean that exposure to the pathogen will lead to either infection or symptoms of disease, but the risk assessment could be extended to include these consequences. We use the definitions for ‘infection’ and ‘disease’ given by [22] i.e. infection is the ‘entrance and development of an infectious agent within a human or animal body’ but does not necessarily lead to disease.

#### 2.1.3. Defining the likelihood pathway

**Figure 1.**
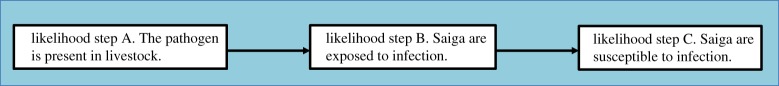
Diagram showing likelihood pathway for the likelihood that saiga will be exposed to livestock pathogens to which they are susceptible.

A likelihood pathway, which consists of a series of likelihood steps (A–C), was constructed (figure 1).

### 2.2. Estimation of likelihood of each likelihood step occurring

The conditional likelihoods of each likelihood step in the likelihood pathway were estimated i.e. (i) the likelihood of A occurring, (ii) the likelihood of B occurring given that A has occurred, and (iii) the likelihood of C occurring given that A and B have occurred. The likelihoods were classified as ‘negligible’, ‘low’, ‘moderate’ or ‘high’, according to the definitions given in table 1.

**Table 1.** Definition of likelihood categories. Likelihood categories and definitions used to classify the likelihood of each likelihood step in the likelihood pathways occurring and to classify the overall likelihood of each likelihood pathway occurring. Categories and definitions taken from [23].

likelihood category	interpretation
negligible	probability of event sufficiently low to be ignored or event only possible in exceptional circumstances
low	occurrence of event is a possibility in some cases
moderate	occurrence of event is a possibility
high	occurrence of event is clearly a possibility

The likelihood estimates were stratified by pathogen, species of domestic livestock (cattle, sheep, goats, camels and horses) and by season, defined as follows (see also electronic supplementary material):

— Spring: 16 March–15 June;
— Summer: 16 June–15th September;
— Autumn: 16 September–15 December; and
— Winter: 16 December–15 March.

The seasons were defined so as to create equal-length time periods that were as distinct as possible in terms of weather, saiga locations and livestock locations.

### 2.3. Combining likelihood steps

The likelihoods of each risk step (A–C) were combined to produce the combined likelihood of Betpak-Dala saiga being exposed and susceptible to a given pathogen, in a given season, via a given livestock species, by taking the lowest of the likelihoods of each step. This is a pre-established method for combining semi-quantitative likelihoods that are dependent on one another [23].

### 2.4. Estimating the likelihood of risk step A: the pathogen is present in livestock

The likelihoods of each pathogen being present in livestock in the rangelands of the Betpak-Dala were classified after, firstly, reviewing official reports from Kazakhstan to the OIE and published literature and, secondly, discussions with veterinarians at the Republican Institute for Biosafety Problems (RIBSP) and the National Reference Centre for Veterinary (NRCV). The individual likelihood classifications for Step A for each pathogen are shown in the supplementary material (table entitled: ‘Combined Likelihood Steps A–C’; column C).

### 2.5. Estimating the likelihood of risk step B: saiga are exposed to infection

Step B (saiga are exposed to infection) was composed of the following conditionally dependent steps:

— B1. There is a specified spatio-temporal interaction between saiga and livestock (see below)
— B2. Saiga are exposed to infection (given the specified interaction).

**Figure 2.**
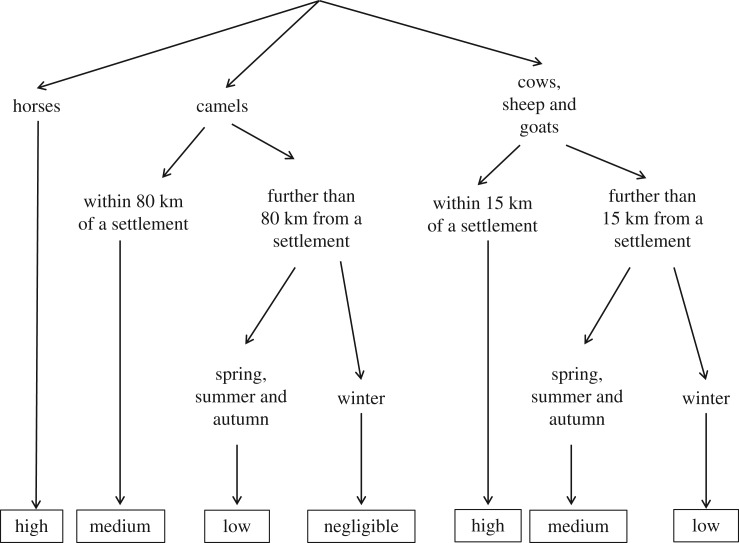
Matrix of possible spatio-temporal interactions between livestock and saiga.

**Figure 3.**
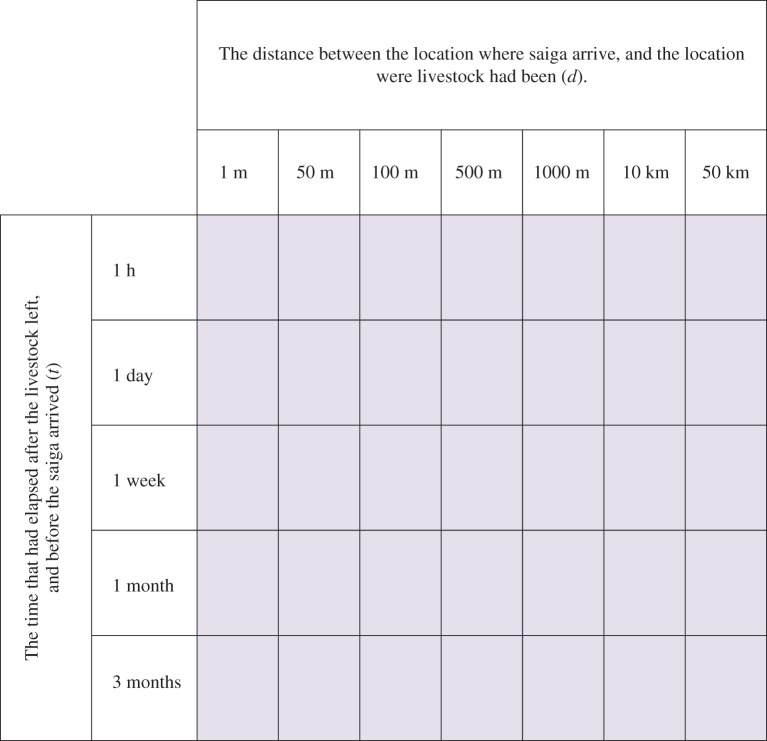
Rules defined to describe likelihoods of various proximities between saiga and livestock in different seasons, based on historical observations.

The specific interactions were defined by a matrix composed of (i) the distance (*d*) between the location where saiga arrive, and the location were livestock had been; and (ii) the time (*t*) that had elapsed after the livestock left, and before the saiga arrived, as shown in figures 2 and 3.

The likelihood estimates for each position in the matrix for Step B1 (the occurrence of a given type of spatio-temporal interaction between saiga and livestock) were completed based on observations of the primary author during field trips in spring 2013 and summer 2015, and semi-structured interviews with livestock owners, wildlife rangers, vets and ecologists during the field trips, based on a pre-designed survey. The survey included questions on when saiga had last been sighted, and if they had observed saiga mixing with livestock. The individual likelihood classifications for Step B1 for each season and for each specific spatio-temporal interaction between saiga and livestock are shown in the electronic supplementary material (table entitled ‘Likelihood Step B’; columns B–I).

The likelihood estimates for each position in the matrix for Step B2 (saiga being exposed to infection given a specified spatio-temporal interaction with infected livestock) were generated via an online survey of experts. The survey consisted of background information on the saiga, its habitat, livestock-keeping practices in the saiga ranges and the climate at different times of year; and questions to elicit the combined likelihood of excretion of pathogen from infected livestock, survival of the pathogen in the environment and exposure of the saiga to the pathogen for each possible spatio-temporal interaction specified in the matrix, for each season.

Experts were first identified using the OIE list of reference laboratories, by disease, and emailed with a link to the pathogen-specific surveys. Personal contacts and authors of publications were then emailed as necessary until there were at least two fully completed surveys for each pathogen. (This was achieved for all pathogens except three, for which only one expert responded: lumpy skin disease virus; *Sarcoptes scabiei* and *Yersinia pseudotuberculosis*.) Likelihood categories were assigned numerical values on a semi-quantitative scale (negligible = 0; low = 1; medium = 2; high = 4), and the final likelihood values used were the mean of the individual experts' likelihood classifications, for each position in the matrix. The final likelihood classifications for Step B2 for each pathogen, season and for each specific spatio-temporal interaction between saiga and livestock are shown in the electronic supplementary material (table entitled ‘Likelihood Step B’; columns K–R). The individual expert likelihood classifications for Step B2 for each pathogen, season and for each specific spatio-temporal interaction between saiga and livestock are shown in the electronic supplementary material (tables entitled: ‘Matrix B2 *pathogen name*’).

Step B was then estimated as follows.

— The resulting matrices for Steps B1 and B2 were combined to produce a single matrix that showed the likelihood of *both* steps occurring, given each possible interaction (Matrix B3) by taking the lowest likelihood for each position in the matrix.
— A single likelihood estimated was generated for Step B, by taking the highest likelihood in the combined matrix (Matrix B3), i.e. the likelihood of the most likely time–distance interaction. The highest likelihood is taken because the different spatio-temporal interactions shown in the matrix are not dependent on one another, but rather any one type of spatio-temporal interaction in the matrix could result in saiga being exposed to the pathogen.

The combined likelihood classifications for Step B for each pathogen and season are shown in the supplementary material (table entitled ‘Combined Likelihood Steps A–C’; column E).

### 2.6. Estimating the likelihood of risk step C: saiga are susceptible to infection

The likelihood of saiga being susceptible to each pathogen was estimated after, firstly, reviewing published literature and, secondly, discussions with veterinarians at the RIBSP and the National Reference Centre for Veterinary (NRCV). The combined likelihood classifications for Step C for each pathogen are shown in the supplementary material (tab entitled ‘Combined Likelihood Steps A–C’; column G).

### 2.7. Uncertainty analysis

Epistemic uncertainty for each likelihood step was classified by the experts (if the expert opinion was sought) or by the authors (if literature review was used) using the definitions shown in table 2. The final uncertainty estimate for each pathway consists of the *highest* of all uncertainty estimates for each risk step, because uncertainty cannot be lower than any one step in the pathway. The process was repeated for each pathogen, season and livestock species, to account for natural variation. The uncertainty classifications are shown alongside each likelihood classification in the electronic supplementary material.

**Table 2.** Definition of uncertainty categories. Uncertainty categories and definitions used to classify the uncertainty associated with the likelihood of each likelihood step in the likelihood pathways, and to classify the overall uncertainty associated with the likelihood of each likelihood pathway occurring. Categories and definitions taken from [23].

uncertainty category	interpretation
low	solid and complete data available; strong evidence provided in multiple references; authors report similar conclusions
medium	some but no complete data available; evidence provided in small number of references; authors report conclusions that vary from one another
high	scarce or no data available; evidence is not provided in references but rather in unpublished reports, based on observations, or personal communication; authors report conclusions that vary considerably between them

### 2.8. Mapping

Likelihood-maps were generated to show where transmission of each disease was most likely in each season, via each livestock species. The maps were constructed by (i) constructing likelihood-maps of livestock locations for each livestock species in each season, and (ii) constructing likelihood-maps of saiga locations in each season. The likelihood categories used were the same as described above (negligible, low, medium or high). The two maps were then combined using the same rule as was used in the combination of likelihood steps above, as the interaction between livestock and saiga is conditional on both being present i.e. the lowest of the two likelihoods was selected. As the final mapping step, the final likelihood-map for each pathogen, livestock species and season was combined with the likelihood pathway estimate for the given pathogen, livestock species and season, using the same combining rule, i.e. for each location on the map, the final likelihood-map displays the combined likelihood of the relevant livestock species and saiga being present, the pathogen being present in livestock, the saiga being exposed to the pathogen, and saiga being susceptible to infection. As livestock census data were only available for some of the saiga distribution area, the study area was reduced to include only areas for which livestock census data were available.

The likelihood-maps for livestock distribution were constructed as follows:

A geodatabase consisting of a shapefile of Selskiy Okrug (rural counties), point locations of settlements and livestock 2008 census data at Selskiy Okrug level in the Betpak-Dala rangelands was obtained from a previous study with permission from the author (Lenk, [24]).

Information about the livestock-keeping practices was obtained through informal discussions and observations during two field trips: a three-week field trip in several villages and the surrounding steppe in the northern area of the Betpak-Dala rangelands in spring 2013 and a three-week field trip travelling along the entire migration route of the Betpak-Dala saiga in July 2015. Based on this information and the authors' own observations, likelihood-maps were constructed based on the rules shown in figure 2.

To create saiga likelihood distribution maps, satellite data from collared saiga collected between September 2009 and March 2015 were transformed as follows:

— A kernel density layer was created from the point locations of saiga, recorded within each season.
— The densities of point locations were transformed into likelihood categories (negligible, low, medium or high) and a contour map was created.
— The contours were transformed into polygons.

### 2.9. Sensitivity analysis

To further assess the effects of epistemic uncertainty and bias on the results, sensitivity analysis was done. Changes were made to the likelihood estimates for each likelihood step, and the resulting final likelihood estimates were compared. This process was conducted using R [25]. For each likelihood step (A–C), and each pathogen, the likelihood estimate was increased by one likelihood category (i.e. from low to medium or medium to high), and the combined likelihood of transmission was re-calculated. The resulting list of pathogens with a combined high likelihood of transmission in any season was compared to the original list, and any changes were recorded. The process was repeated with a decrease for each risk estimate. In total, 408 individual changes to risk estimates were tested (68 for risk steps A and C; and 68 for each of four seasons for risk step B).

## 3. Results

One-hundred-and-seventeen pathogens featured on the OIE list of livestock diseases, in addition to 45 on the OIE list of wildlife diseases. Of these, 33 satisfied the inclusion criteria.

The following pathogens had a negligible risk of transmission in all seasons: Aujeszky's disease virus, *Echinococcus granulosus* and *Trichinella* spp. The seven most likely pathogens predicted to be transmitted were *Brucella* spp., *Chlamydophila abortus*, foot-and-mouth disease virus, *Leptospira interrogans*, *Listeria monocytogenes*, *Salmonella enterica* and *Theileria ovis*. The full list of pathogens included is in the electronic supplementary material.

**Figure 4.**
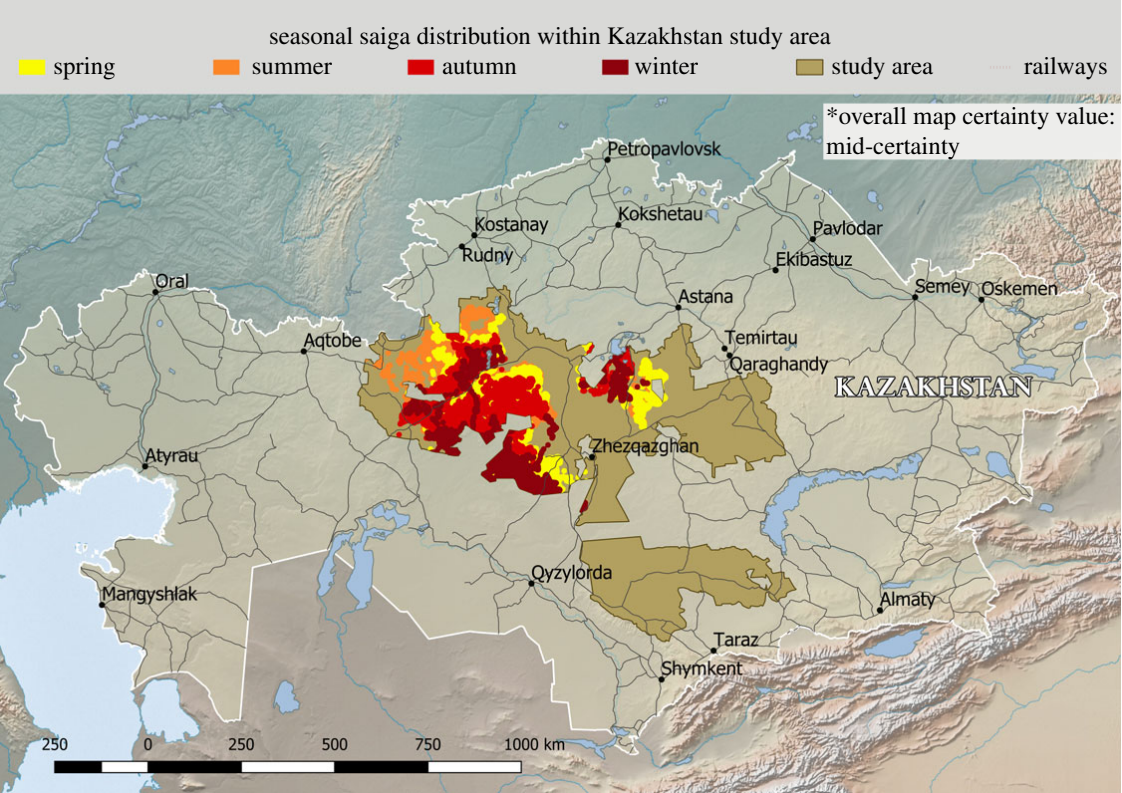
Maps showing risk assessment study area and the saiga presence in (*a*) spring; (*b*) summer; (*c*) autumn and (*d*) winter, based on satellite collar data collected in 2009–2015.

**Figure 5.**
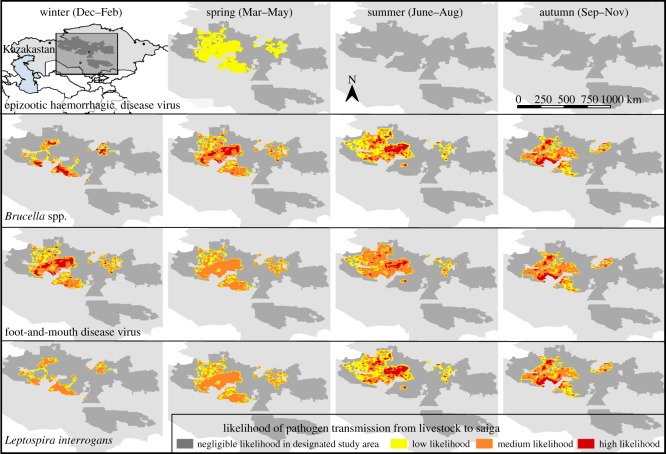
Maps of the study area in rangelands of Betpak-Dala saiga showing likelihood of saiga being exposed and susceptible to different livestock pathogens.

Figure 4 shows the study area and the geolocations where saiga were assumed to be present in each season based on satellite collar data of saiga in 2009–2015. Figure 5 shows the areas where pathogen transmission from livestock to saiga was classified as high, medium, low or negligible by season, for four selected pathogens. The overall uncertainty associated with the final likelihood-maps for each pathogen was as follows: *Brucella* spp.—medium; epizootic haemorrhagic disease virus—high; foot-and-mouth disease virus—medium; *Leptospira interrogans*—medium. Low to high uncertainty was associated with all three likelihood steps (A–C), depending on the pathogen, and medium uncertainty was associated with the geolocations of livestock and saiga. The maps for each pathogen with at least a low likelihood are included in the electronic supplementary material.

A single-category increase in each likelihood estimate for likelihood step A did not result in any further pathogens being added to the list of pathogens with a high combined likelihood of exposure and susceptibility. A single-category decrease (high to medium, medium to low, or low to negligible) in each likelihood estimate for likelihood step A resulted in none of the pathogens having a combined high likelihood of exposure and susceptibility.

A single-category increase in each likelihood estimate for likelihood step B, in each season, resulted in the following pathogens being added to the list of pathogens with a high combined likelihood of exposure and susceptibility: *Bacillus anthracis*, nematodes, *Mycobacterium tuberculosis* subsp. paratuberculosis and *Psoroptes* spp. A single-category decrease in each likelihood estimate for likelihood step B was assessed separately for each season and had no impact on the final list of pathogens with a combined high likelihood of exposure and susceptibility in any season.

A single-category increase in each likelihood estimate for likelihood step C resulted in *Clostridium piliforme* being added to the list of pathogens with a high combined likelihood of exposure and susceptibility. A single-category decrease in each likelihood estimate for likelihood step C resulted in none of the pathogens having a combined high likelihood of exposure and susceptibility.

## 4. Discussion

In this study, we have not only demonstrated a systematic method for identifying the specific pathogens most likely to spread from livestock to saiga, but also where and when this is most likely to occur, thus creating a model for future risk assessments of disease transmission between domestic animals and wildlife.

In summary, of 33 pathogens of concern the seven most likely pathogens predicted to be transmitted were: *Brucella* spp., *Chlamydophila abortus*, foot-and-mouth disease virus, *Leptospira interrogans*, *Listeria monocytogenes*, *Salmonella enterica* and *Theileria ovis*. Brucellosis and foot-and-mouth disease were reported many years ago in saiga, although not recently. However, the published literature is likely to over-represent brucellosis and foot-and-mouth disease, as these are important diseases from an economic and public health perspective. Morgan *et al*. [19] explored the use of a compartmental transmission model to predict the consequences of saiga herds becoming infected with foot-and-mouth disease, in terms of the spread of infection within the herd. Although useful for investigating the likely importance of seasonality, for example, the model cannot be reliably used to predict the overall likelihood of transmission, as the transmission parameter was not validated with field data.

We found no references to *Chlamydophila abortus*, *Leptospira interrogans*, *Listeria monocytogenes* or *Salmonella enterica* infections in saiga. However, these pathogens are common in ruminant species worldwide, and it is likely that saiga could be infected, as many of these pathogens have been identified in wild deer populations. It is also of note that many of these pathogens can be transmitted to humans.

The areas where transmission was most likely to occur were relatively small, as would be expected as saiga avoid populated areas and we assumed livestock densities were low beyond a certain distance of the populated areas. This suggests saiga could be naive to livestock pathogens and therefore lack protective immunity. This could make them susceptible to sporadic pathogen introductions, resulting in epidemics. This is a pattern that has been observed historically in saiga populations, for example, the recent peste des petits ruminants outbreak in Mongolia [14,26]. This suggests that surveillance of both livestock and saiga distributions, and any likely overlap in distributions, are important in addition to monitoring of saiga population health. In addition, it should be ensured there are incentives for livestock owners and veterinarians to report relevant disease events, and that there are no negative consequences for doing so.

One strength of our approach is that it can be used when there is very little documented evidence—in fact, it could be done solely relying on the opinions of experts. In situations where there are no objective data available, it is a useful way of making systematic judgements for the purposes of decision-making. However, the opinions of experts need to be gathered systematically to reduce bias as far as possible, and the subjectivity of expert opinion needs to be acknowledged. These concerns can be mitigated by following rigorous methods for gathering expert opinion. There are uncertainties that cannot be addressed by risk assessment, such as pathogens or transmission mechanisms that are not yet discovered. For these reasons, it is important that the uncertainties associated with the risk assessment outputs are explicitly expressed, and an uncertainty analysis is done.

Important sources of uncertainty were associated with all three likelihood steps (A–C) and the geolocations of livestock and saiga. A particularly important source of uncertainty was that the livestock location maps were based on census data, and it is possible that livestock is grazed remotely from their owners, or that populations have changed since the census took place. Betpak-Dala saiga geographical distributions also vary from year to year, and have gradually moved further north over the last several decades. The maps were also generated based on the locations of only a proportion of the total Betpak-Dala saiga population.

There was, as expected, some disagreement between experts in the overall individual likelihood classifications given in the matrices although the overall expectations of seasonal and spatio-temporal effects were subjectively similar. In addition, for three pathogens, only one expert responded to the survey. Sensitivity analysis suggested that changes to individual assessments of each step had a relatively minimal effect on the overall conclusion of which pathogens were classified as the high likelihood in terms of saiga being exposed. This suggests that this model framework was fairly robust to uncertainties in the individual likelihood steps, despite the lack of data available.

For practical reasons, the model framework requires that certain limits are placed on the number of scenarios that are assessed. We selected only infectious organisms that were included in either the OIE list of wildlife diseases or the OIE-listed livestock diseases, and have therefore not considered some organisms that could be relevant to saiga conservation. As part of the model framework, we only considered transmission that could occur up to three months after pathogens were shed into the environment. This upper limit was chosen because each season (defined as three-month periods) was assessed separately, and because it was considered a reasonable period of time for experts to classify likelihoods of pathogen survival. It is a limitation of this approach that a pathogen that persists for longer than three months in the environment may still be spread from livestock to saiga beyond three months after livestock were present in a given location. This scenario was not explicitly included in the risk assessment. However, in most cases, the likelihood of transmission can be expected to diminish after the pathogen has been present in the environment for longer than three months, firstly because the pathogen would be less likely to survive, and secondly because saiga are less likely to be present in the same area as livestock beyond three months (for climatic reasons).

The risk assessment framework we propose could be applied to any context where pathogens may spread between two populations, and especially when established resources or data are minimal. It can be applied to species with complex migratory patterns and can serve as a useful tool for specialists in one area of wildlife disease or conservation to gather and synthesize critical pieces of information from other fields with which they may be less familiar. It can also be used to identify key knowledge gaps and can be updated as further information becomes available.

## Supplementary Material

Pathogens that satisfied the inclusion criteria.

## Supplementary Material

Likelihood maps for transmission of pathogens from domestic livestock to the Betpak Dala saiga population.

## Supplementary Material

Likelihood and uncertainty classifications for each likelihood step and pathway

## Data accessibility

The datasets supporting this article have been uploaded as part of the electronic supplementary material.
