# Supplementary material for: Rapidly assessing the risks of infectious diseases to wildlife species
Source: R Soc Open Sci. 2019 Jan 16;6(1):181043. doi: 10.1098/rsos.181043 (PMC6366200; doi:10.1098/rsos.181043)
Supplement: Pathogens that satisfied the inclusion criteria. [file rsos181043supp1.docx]

| **Viruses** | **Bacteria** | **Parasites** |
| --- | --- | --- |
| Aujesky's disease virus | *Anaplasma marginale* | *Echinococcosis granulosus* |
| Bluetongue virus | *Babesia bigemina, B. bovis, B. divergens* and *B. ovis* | *Fasciola gigantica and magna* |
| Bovine viral diarrhoea virus | *Bacillus anthracis* | *Psoroptes* spp. |
| Crimean Congo haemorrhagic fever virus | *Brucella abortus* and *B. melitensis* | *Sarcoptes scabeii* |
| Epizootic haemorrhagic disease virus | *Chlamydophila abortus* | *Trichinella spp.* |
| Flavivirus (tick borne encephalitis) | *Clostridium piliforme* (Tyzzer's Disease) |  |
| Foot-and-mouth disease virus | *Leptospira interrogans* |  |
| Infectious bovine rhinotracheitis virus | *Listeria monocytogenes* |  |
| Low pathogenic avian influenza virus | *Mycobacterium bovis* |  |
| Lumpy skin disease virus | *Mycobacterium tuberculosis subsp.* paratuberculosis |  |
| Malignant catarrhal fever virus (OHV2) | *Mycoplasma capricolum* subsp*.* capripneumoniae  (Contagious caprine pleuropneumonia) |  |
| Peste des petits ruminants virus | *Pasteurella multocida* and other *Pasteurella spp.* |  |
| Rabies virus | *Salmonella enterica* |  |
| Sheep pox and goat pox viruses |  |  |
