# Supplementary material for: Rapidly assessing the risks of infectious diseases to wildlife species
Source: R Soc Open Sci. 2019 Jan 16;6(1):181043. doi: 10.1098/rsos.181043 (PMC6366200; doi:10.1098/rsos.181043)
Supplement: Likelihood maps for transmission of pathogens from domestic livestock to the Betpak Dala saiga population. [file rsos181043supp2.pdf]

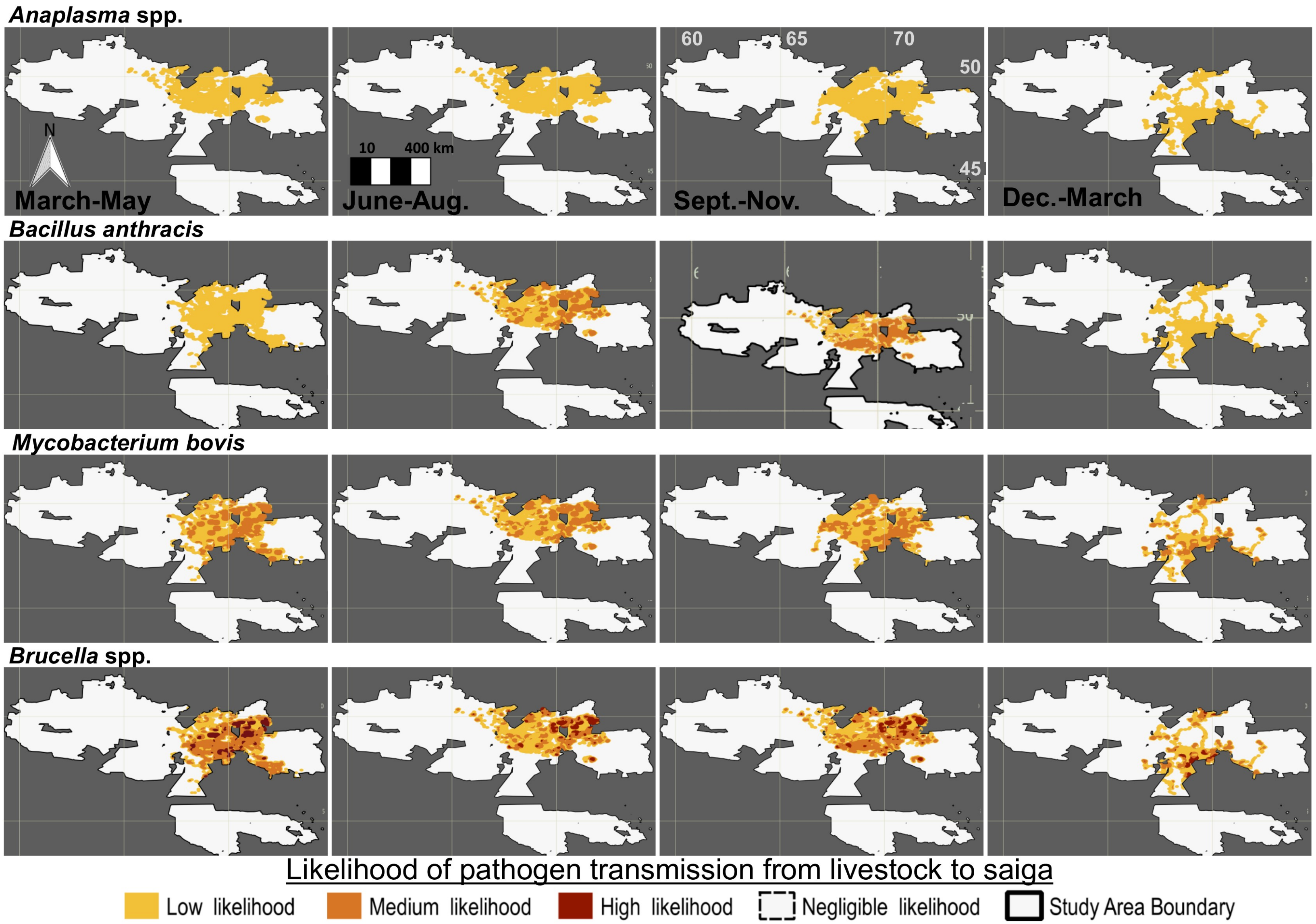

*Anaplasma* spp.
*Bacillus anthracis*
*Mycobacterium bovis*
*Brucella* spp.
March-May
June-Aug.
Sept.-Nov.
Dec.-March
N
10
400 km
60
65
70
50
45
Likelihood of pathogen transmission from livestock to saiga
Low likelihood
Medium likelihood
High likelihood
Negligible likelihood
Study Area Boundary

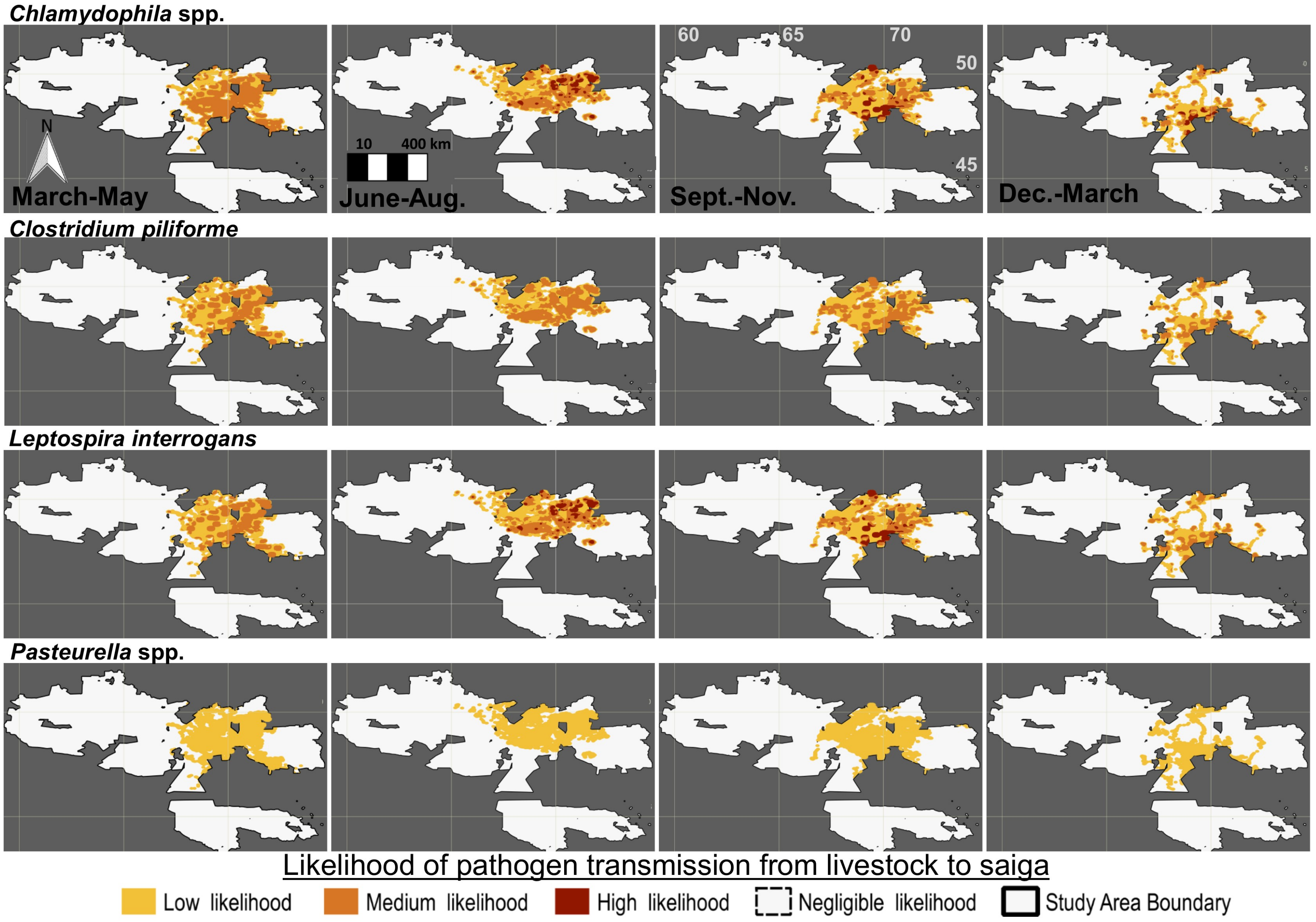

Chlamydophila spp.
60
65
70
50
45
N
10
400 km
March-May
June-Aug.
Sept.-Nov.
Dec.-March
Clostridium piliforme
Leptospira interrogans
Pasteurella spp.
Likelihood of pathogen transmission from livestock to saiga
Low likelihood
Medium likelihood
High likelihood
Negligible likelihood
Study Area Boundary

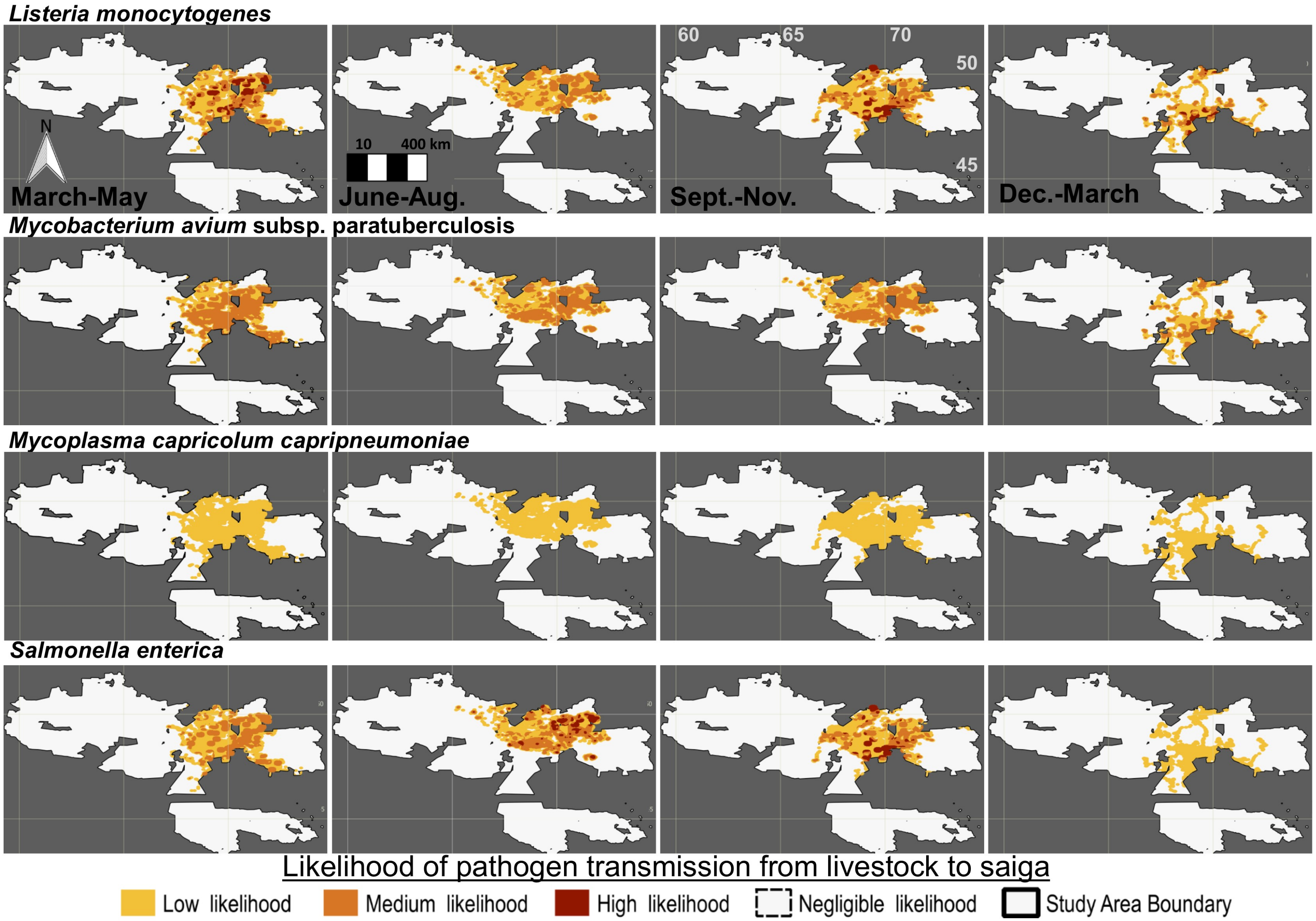
Listeria monocytogenes
60
65
70
50
45
N
10
400 km
March-May
June-Aug.
Sept.-Nov.
Dec.-March
Mycobacterium avium subsp. paratuberculosis
Mycoplasma capricolum capripneumoniae
Salmonella enterica
Likelihood of pathogen transmission from livestock to saiga
Low likelihood
Medium likelihood
High likelihood
Negligible likelihood
Study Area Boundary

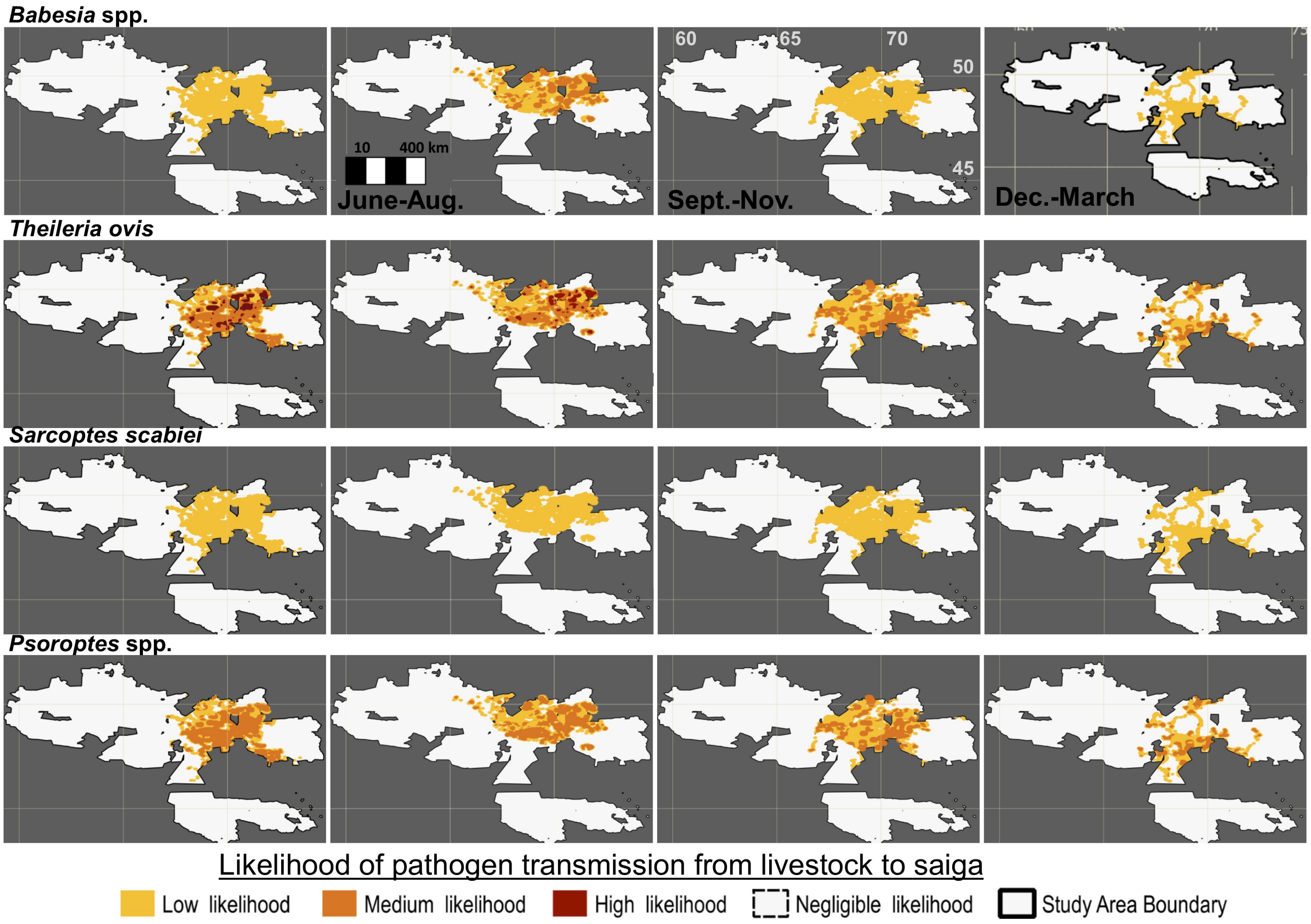

Babesia spp.
Theileria ovis
Sarcoptes scabiei
Psoroptes spp.
10
400 km
June-Aug.
Sept.-Nov.
Dec.-March
60
65
70
50
45
Likelihood of pathogen transmission from livestock to saiga
Low likelihood
Medium likelihood
High likelihood
Negligible likelihood
Study Area Boundary

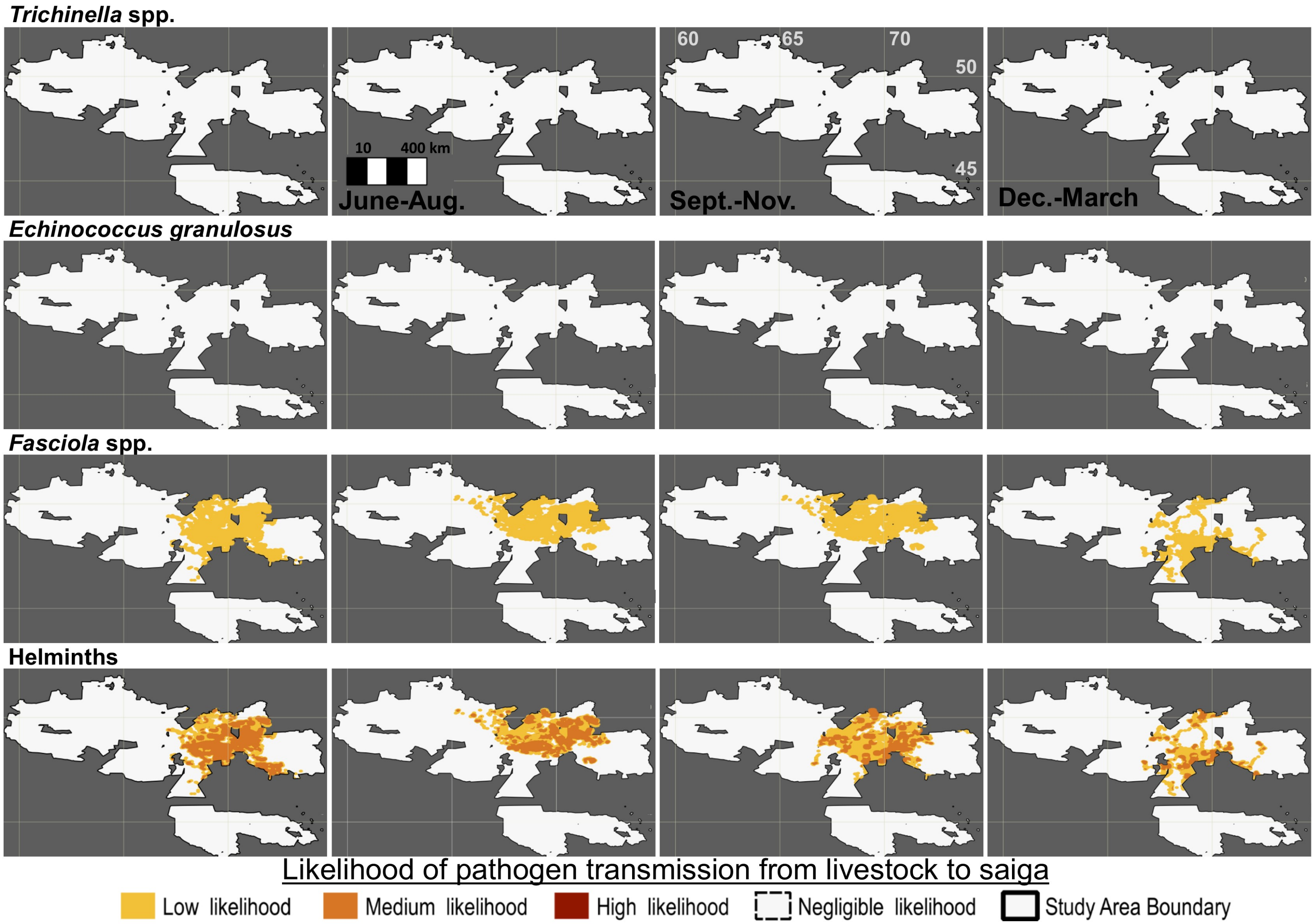
Trichinella spp.
60
65
70
50
45
10
400 km
June-Aug.
Sept.-Nov.
Dec.-March
Echinococcus granulosus
Fasciola spp.
Helminths
Likelihood of pathogen transmission from livestock to saiga
Low likelihood
Medium likelihood
High likelihood
Negligible likelihood
Study Area Boundary

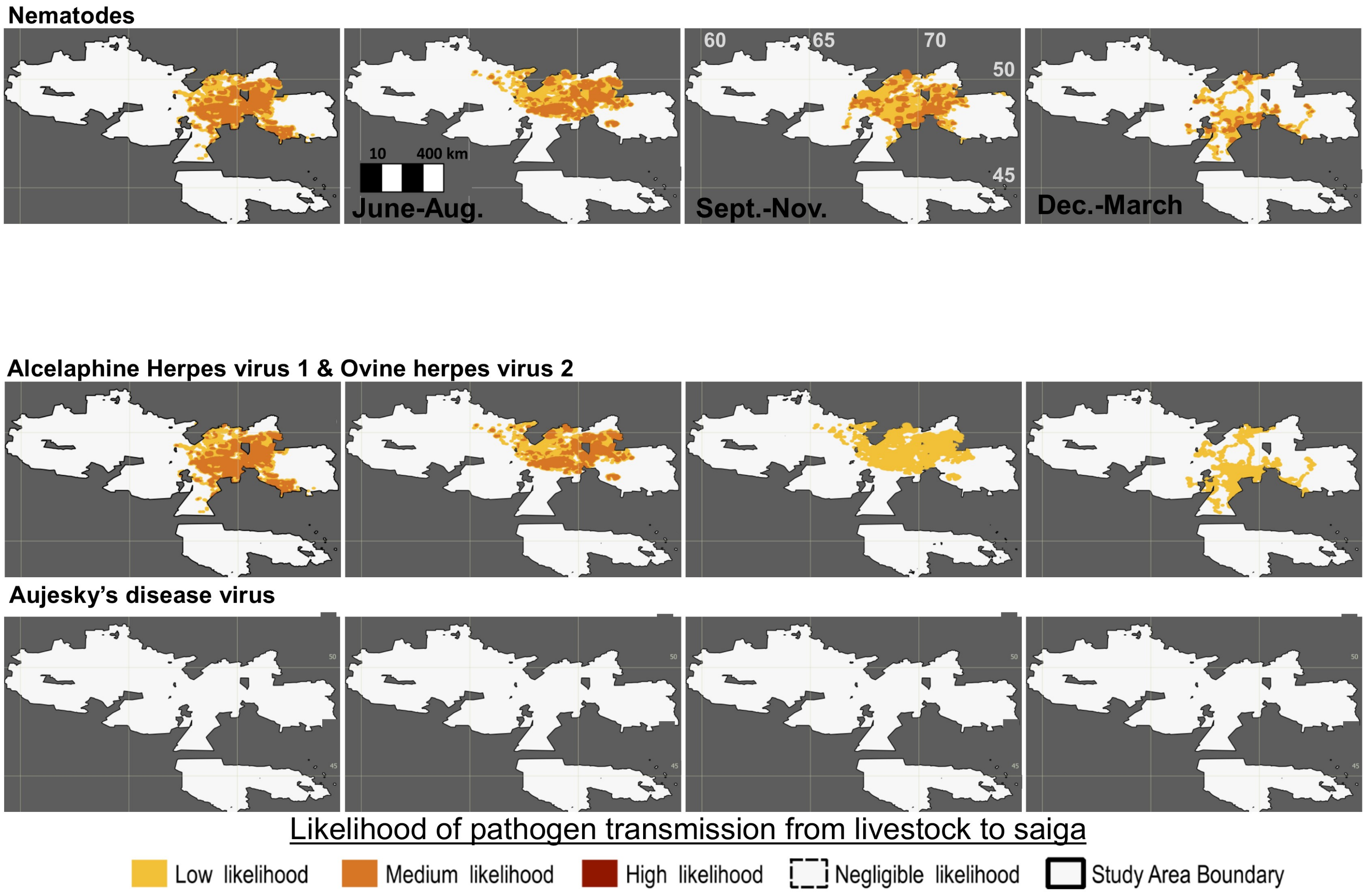

Nematodes
10
400 km
June-Aug.
60
65
70
50
45
Sept.-Nov.
Dec.-March
Alcelaphine Herpes virus 1 & Ovine herpes virus 2
Aujesky's disease virus
Likelihood of pathogen transmission from livestock to saiga
Low likelihood
Medium likelihood
High likelihood
Negligible likelihood
Study Area Boundary

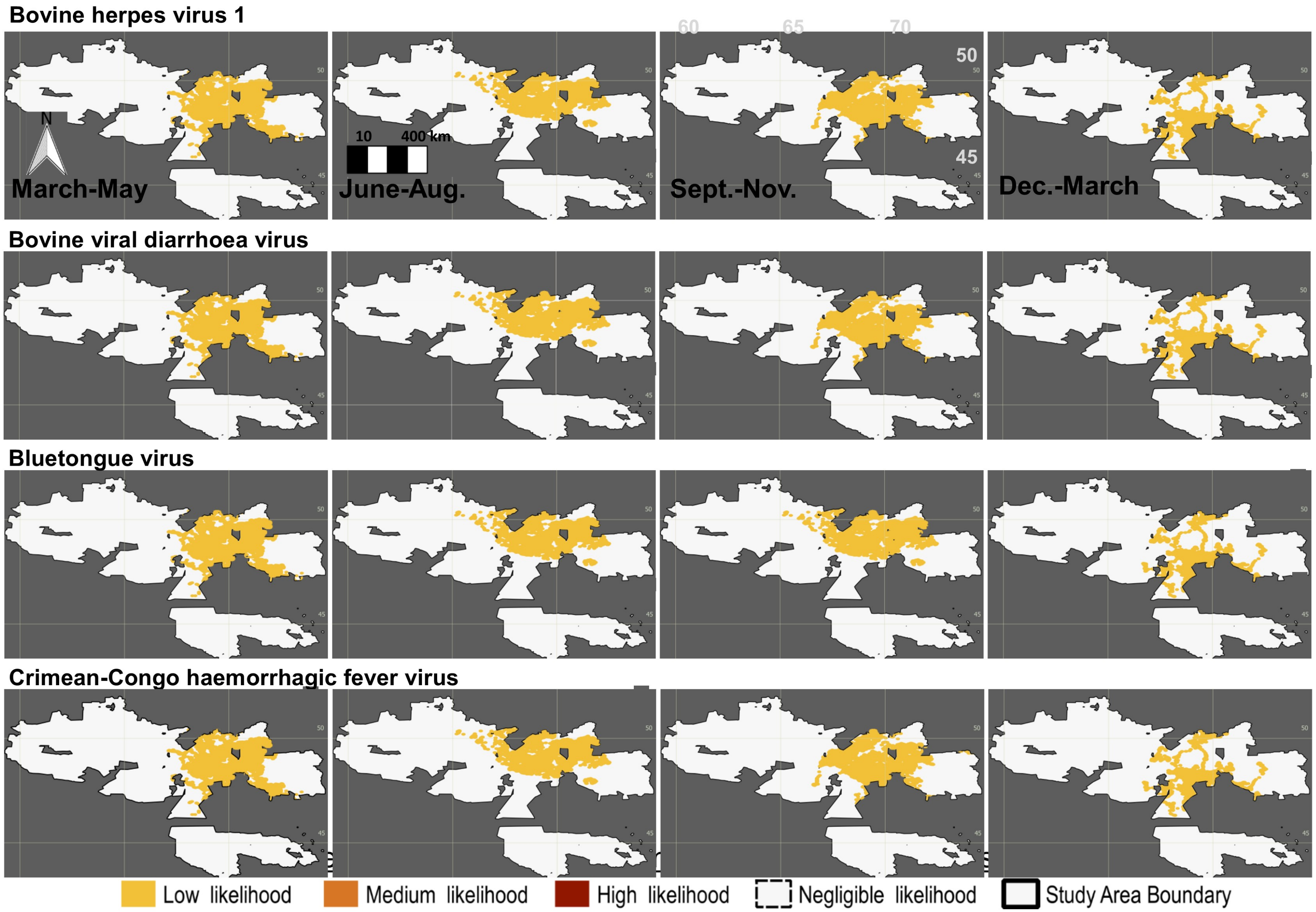
Bovine herpes virus 1
March-May
June-Aug.
Sept.-Nov.
Dec.-March
N
10
400 km
60
65
70
50
45
Bovine viral diarrhoea virus
Bluetongue virus
Crimean-Congo haemorrhagic fever virus
Low likelihood
Medium likelihood
High likelihood
Negligible likelihood
Study Area Boundary

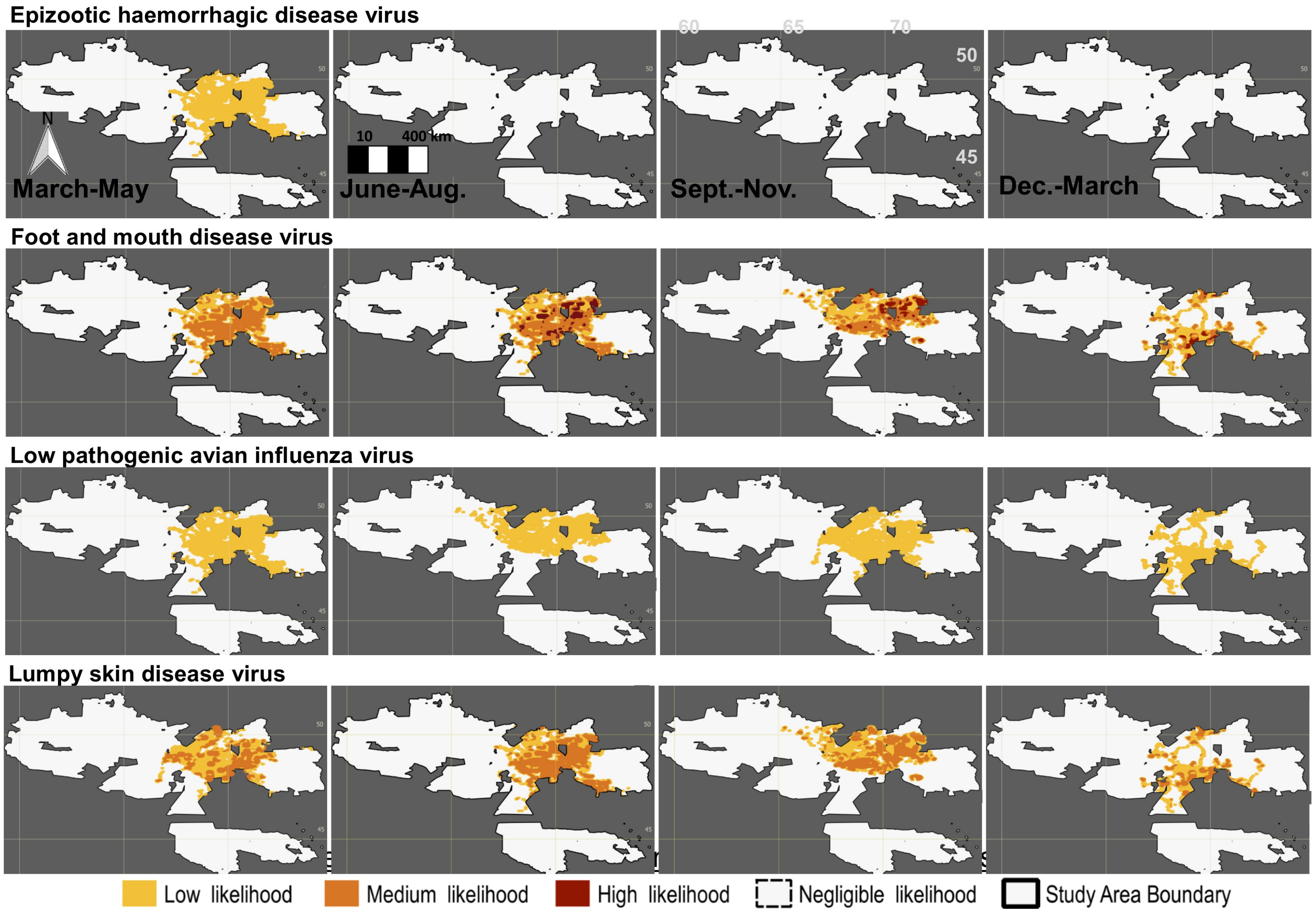

Epizootic haemorrhagic disease virus
60
65
70
50
45
N
10
400 km
March-May
June-Aug.
Sept.-Nov.
Dec.-March
Foot and mouth disease virus
Low pathogenic avian influenza virus
Lumpy skin disease virus
Low likelihood
Medium likelihood
High likelihood
Negligible likelihood
Study Area Boundary

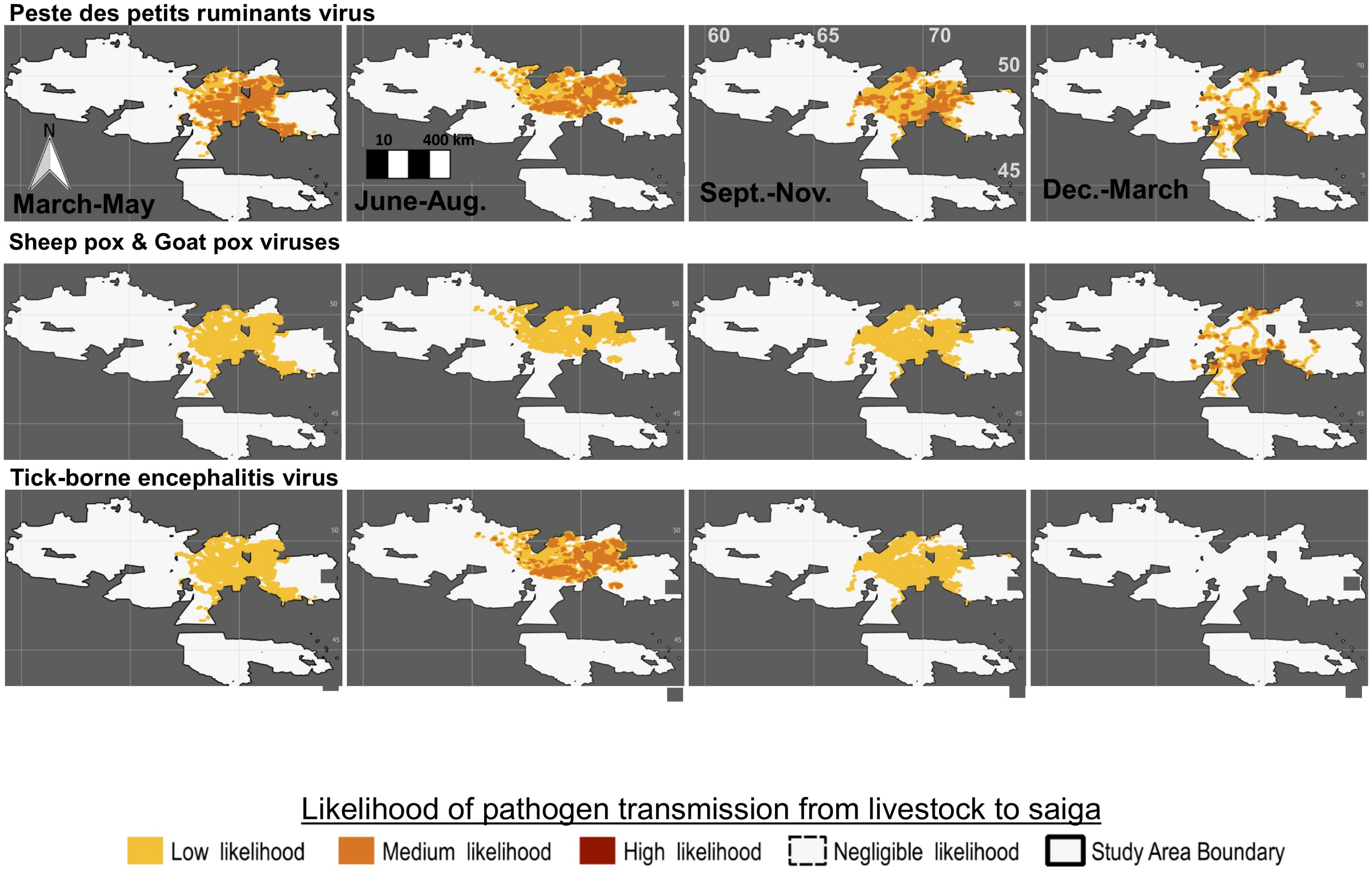

Peste des petits ruminants virus
March-May
June-Aug.
Sept.-Nov.
Dec.-March
N
10
400 km
60
65
70
50
45
Sheep pox & Goat pox viruses
Tick-borne encephalitis virus
Likelihood of pathogen transmission from livestock to saiga
Low likelihood
Medium likelihood
High likelihood
Negligible likelihood
Study Area Boundary
